# Inhibitors and facilitators of compliance with professional ethics standards: nurses’ perspective

**DOI:** 10.1186/s12912-024-01829-9

**Published:** 2024-03-05

**Authors:** Shahram Sharifi, Hossein Ebrahimi, Shahla Elyaszadeh, Arman Latifi, Mohammad Taghi Khodayari, Maedeh Alizadeh

**Affiliations:** 1 Student Research Committee, Maragheh University of Medical Sciences, Maragheh, Iran; 2 Department of Psychiatric Nursing, Nursing and Midwifery Faculty, Tabriz University of Medical Sciences, Tabriz, Iran; 3 Student Research Committee, Tabriz University of Medical Sciences, Tabriz, Iran; 4 Department of Public Health, School of Public Health, Research Center for Evidence Based Health Management, Maragheh University of Medical Sciences, Maragheh, Iran; 5 Research Center for Evidence-Based Health Management, Maragheh University of Medical Sciences, Maragheh, Iran

**Keywords:** Codes of Ethics, Ethics, Professional, Nursing

## Abstract

**Introduction:**

The clinical practices of nurses should be in accordance with the principles of professional ethics. Respecting professional ethics principles depends on several factors. The present study was conducted to investigate the effective inhibitors and facilitators in compliance with professional ethics and their importance from the nurses’ perspective.

**Methods:**

During this cross-sectional descriptive study, 452 nurses were included by the census sampling method. The data were collected via the “inhibitors of compliance with professional ethics standards by the nurses’ perspective” and “facilitators of compliance with professional ethics standards by the nurses’ perspective” questionnaires. Additionally, by designing the “open-ended question” section, other inhibiting and facilitating factors of professional ethics standards from the nurses’ perspective were investigated. The data were analyzed using descriptive and inferential statistics (Wilcoxon signed ranks test).

**Results:**

The individual care-related dimension as a facilitator had the highest mean score compared to the other dimensions (76.62 ± 4.92). Furthermore, seven items in the inhibitor section, 19 items in the facilitator section had higher scores. Among them, there were seven items in common. Strong or weak belief in compliance with ethical issues had the highest mean scores in the role of facilitator and inhibitor (90.54 ± 12.13 and 89.54 ± 14.88, respectively).

**Conclusion:**

Strong or weak belief in compliance with ethical issues was the most important inhibitor and facilitator from the nurses’ perspective, which makes it necessary to examine individual beliefs about ethical issues among applicants to enter the nursing profession.

**Supplementary Information:**

The online version contains supplementary material available at 10.1186/s12912-024-01829-9.

## Introduction

Receiving health services is one of the most important foundations of human rights; in this regard, the nursing profession, with maximum care professionals, is responsible for providing health services at the highest standard level [1]. From Henderson’s point of view, the unique work of nurses in providing healthcare services and acquiring moral knowledge is next to physiological knowledge. Therefore, the clinical practice and care of nurses are associated with ethics [2], and commit nurses to comply with professional ethics at all levels of care [3].

Professional ethics for nurses is based on professional and personal commitment, which includes the three dimensions of rights, duties, and responsibilities. These regulate the behavior of nurses in relation to patients, colleagues, other professions, and the organization and aim to protect patients and guide nurses to help promote a healthy society. In fact, with respect the standards of professional ethics, nurses undertake to guide their professional activities to protect the client from harm and benefit from care [4].

Despite the emphasis on compliance with professional ethics standards (PES), studies show that the level of compliance with professional ethics among nurses is not ideal and nurses perform poorly in terms of commitment, confidentiality, privacy, responsibility, and professional skills [5, 6]. Based on this, nurses consider inhibitors and facilitators to be involved in compliance with professional ethics [3]. In their view, lack of ethics retraining courses, inappropriate communication between nurses and head nurses, lack of suitable equipment are inhibitors [7], and individual beliefs and the promotion of ethical knowledge are facilitators of compliance with PES [8].

In the study of Esmaelzadeh et al., “the ability of nurses to make ethical decision” as a facilitating factor in compliance with PES [9], and in the study s of Khaki et al., “inability of nurses to make ethical decisions” as an inhibiting factor in compliance with PES from the point of view of nurses have been reported [7]. At first glance, it seems that the presence of a factor in the observance of PES as a significant facilitator can indicate the importance of its absence as an inhibitor. This is despite the fact that in the study of Saeeidi et al., “the strength of the religious and beliefs of nurses” is considered as an important facilitator in compliance with PES [8], but in the study of Dehghani et al., “weakness of religious and beliefs of nurses” was not mentioned as an important inhibitor in compliance with professional ethics [10]. Also, in the study of Jafari et al., fixed work shifts and the existence of appropriate interpersonal communication were found to facilitate the observance of professional ethics, while in the study of Wilson Barnett, rotating shifts were considered as an inhibitor to the observance of professional ethics. Additionally, in Dehghani et al.‘s study, from the nurses’ perspective, lack of proper interpersonal communication was not the most important obstacle [10–12].

According to the results of previous studies, the question of which side of the facilitating and inhibiting spectrum nurses give more importance to in compliance with PES is in an aura of uncertainty. One of the reasons could be the lack of simultaneous investigation of the inhibitors and facilitators of compliance with PES in the same study. Therefore, there are doubts about the importance of the factors involved in compliance with or not with professional ethics from the perspective of nurses. Therefore, it seems that the determination of important inhibitors and facilitators can be helpful in planning management programs to promote compliance with PES. Based on this, the present study was conducted to investigate the effective inhibitors and facilitators in compliance with professional ethics and their importance from the nurses’ perspective.

## Methods

### Design and setting

The current study is a cross-sectional descriptive study that was conducted with the aim of determining the importance of effective facilitating and inhibiting factors in compliance with PES from the nurses’ perspective. Information was collected from medical and educational centers and hospitals affiliated with the Maragheh Faculty of Medical Sciences, Maragheh, Iran, including Amir al-Momenin, Sina, and Shahid Beheshti.

### Participants

Among 470 nurses working in the medical centers of Maragheh, 452 nurses were included by census sampling method. The inclusion criteria were as follows: informed consent to participate in the study, having a bachelor’s degree in nursing or above, and at least six months of experience. Nurses who stopped completing the questionnaire after entering the study were excluded from the study.

### Instrument and data collection

For data collection, a three-part paper-based questionnaire was used. The first part included demographic information: age, gender, marital status, education level, work shift, and nursing experience. The second part included a questionnaire on facilitating and inhibiting factors of PES from the nurses’ perspective. The third part was designed as an open-ended question, and the participants were asked to share with the research team other factors of compliance with professional ethics that were not mentioned in the questionnaire.

In this study, the questionnaire “Inhibitors of compliance with professional ethics standards by the nurses’ perspective” developed by Dehghani et al. was used to investigate the inhibitors of compliance with PES. This questionnaire contains 33 items on inhibitors of compliance with the PES from the nurses’ perspective. The items in this questionnaire are placed in 3 dimensions: management (14 items), individual care-related (14 items), and environmental (5 items). Each item in this questionnaire has a negative meaning that its answers are on a Likert scale (completely agree, agree, no opinion, disagree, and strongly disagree) [10]. In this study, we set the scoring of each item between 0 and 100. A score closer to 100 indicates the high importance of non-compliance with PES from the nurses’ perspective. The reliability and validity of this questionnaire have been confirmed by using the content validity index (CVI) and Cronbach’s alpha coefficient (0.89) [10].

Given that we wanted to know which role (inhibition or facilitation) of effective factors in compliance with professional ethics is more important from the nurses’ perspective, we designed a questionnaire titled “Facilitators of compliance with professional ethics standards by the nurses’ perspective” according to the “Inhibitors of compliance with professional ethics standards by the nurses’ perspective” [10]. For this purpose, we replaced all of the items in Dehghani et al.‘s questionnaire [10] with positive meaning phrases and then used it as the questionnaire of “Facilitators of compliance with professional ethics standards by the nurses’ perspective”. The dimensions and methods of scoring the items of this questionnaire were similar to those of the “inhibitors of compliance with professional ethics standards by the nurses’ perspective” questionnaire. A score closer to 100 indicates the high importance of compliance with PES from the nurses’ perspective.

The validity of the questionnaire was determined by the content validity index (CVI) and the content validity ratio (CVR). For this purpose, the questionnaire was assessed by 10 experts in professional ethics in nursing, including three nurses with more than 10 years of experience who have a certificate of attendance at the professional ethics workshop and 7 faculty members of nursing who were lecturers of professional ethics. Then, the simplicity, clarity, and relevancy of each item were assessed by using the “Waltz & Bausell” method with scores ranging from 1 to 4. Also, the CVR was assessed by asking questions about the necessity of each item in a three-point Likert scale (necessary, useful but not necessary, and not necessary) using Lawshe’s method. In total, the CVI and CVR were 0.80 and 0.68, respectively, in our study. To determine the internal consistency reliability of the questionnaires, in a pilot study, 30 nurses completed both the inhibitors and facilitators of compliance with PES questionnaires, and the Cronbach’s alpha coefficient was calculated 0.89 and 0.87, respectively.

The third part of the questionnaire included an open-ended question. In this section, the participants were asked to answer the following section: ‘’Please share with the research team other factors of compliance with professional ethics that were not mentioned in the questionnaire”. Participants’ answers in this section were checked and then summarized and reported.

The data were collected from May to December 2022. After the study objectives and methods were explained to the nurses and informed consent was obtained, the nurses completed the questionnaires. Completing each questionnaire took approximately 25 to 30 min.

### Statistical analyses

The data were analyzed using inferential and descriptive statistics. The Wilcoxon signed-rank test was used to compare the mean scores of the same dimensions and items for the two inhibitors and facilitators of compliance with the PES questionnaires. A significance level of less than 0.05 was considered. Data analysis was performed using SPSS 16 software.

## Results

### Participant characteristics

The average age of the participants was 31.35 ± 4.54 years, ranging from 23 to 51 years, and they were working as clinical nurses, supervisors, and matrons. Other demographic characteristics of the participants are shown in Table 1.

The mean scores of the dimensions and items are presented in Table 2. Accordingly, the individual care-related dimension as a facilitator had the highest mean score compared to the other dimensions (76.62 ± 4.92). Furthermore, comparing the mean scores of the facilitating and inhibiting dimensions and items using the Wilcoxon signed-rank test is also shown in this table.

Based on the mean score of the items, seven items in the inhibitor section and 19 items in the facilitator section had higher and closer mean scores to 100. Among them, there were seven items in common, which are shown in Table 2.

### Other inhibitors and facilitators

A total of 183 nurses (40%) out of 452 responded to the “open-ended question” section about “other factors that facilitate and inhibit compliance with nurses’ professional ethics standards”, and each of them mentioned one to three items, which included too many work shifts (*n* = 85), fatigue by working with a mask (*n* = 32), lack of encouragement and punishment criteria for compliance with professional ethics standards (*n* = 72), lack of peace in the work environment (*n* = 37), and empathy and compassion for patients (*n* = 42).

**Table 1:** Demographic characteristics of participants

Demographic variables		Mean (SD)
Age (year), Mean (SD)		31.35 (4.54)
Total work experience (years)		7.84 (3.75)
Work experience in the current Ward (years)		4.04 (1.679)
N (%)		
Gender	Female	329 (72.8)
	Male	123 (27.2)
Marital status	Single	124 (27.4)
	Married	328 (72.6)
Ward	Internal medicine	158 (35)
	surgery	208 (46)
	ICU	13(2.9)
	CCU	37 (8.2)
	Other	37 (8.2)
Position	General Nurse	444 (98.2)
	Other	8 (1.8)
Shifts	Fixed morning	81 (17.9)
	Rotating	371 (82.1)

Note: SD: Standard Deviation

**Table 2:** The mean scores of the inhibitors and facilitators to compliance with PES by the dimensions

Dimension and Item	Inhibitors to compliance with PES Mean (SD)	Facilitators to compliance with PES Mean (SD)	***p*** value ^a^
**Individual care-related dimension**	56.51(8.39)	76.62 (4.92)	< 0.001
*Strong belief in compliance with ethical issues *Weak belief in compliance with ethical issues	89.54 (14.88)	90.54 (12.13)	0.401
*Having knowledge and awareness about the principles of professional ethics *Not having knowledge and awareness about the principles of professional ethics	82.07 (15.95)	87.38 (12.51)	< 0.001
*Low workload and its impact on accuracy and concentration of nurses *High Workload and its impact on accuracy and concentration of nurses	80.69 (24.05)	87.11 (16.51)	< 0.001
*Ability to critical thinking or ethical decision-making *Inability to critical thinking or ethical decision-making	86.06 (20.50)	87.05 (12.50)	0.853
*Effective and appropriate communication with the patient *In effective and inappropriate communication with the patient	80.36 (21.32)	83.24 (19.26)	0.173
*Responding to basic needs such as income sufficiency or adequate rest in nursing staff Failure to respond to basic needs such as adequate income or rest in nursing staff	56.08 (35.30)	87.83 (12.50)	< 0.001
*Satisfaction with the work place Dissatisfaction with the work place	17.86 (23.07)	87.72 (15.83)	< 0.001
*Having enough time required to perform care Not having enough time required to perform care	75.27 (32.08)	81.74 (19.73)	< 0.001
*Interest and motivation toward the profession in nursing personnel Lack of interest and motivation to the profession in nursing personnel	65.54 (33.09)	80.25 (13.37)	< 0.001
*Sufficient previous familiarity with the skills Carrying out completely new tasks without previous familiarity and skills	36.06 (34.23)	79.92 (25.93)	< 0.001
*Positive attitude to professional ethics standards in nursing Negative attitude to professional ethics standards in nursing	42.92 (35.74)	79.20 (16.69)	< 0.001
Cooperation of patients Failure to cooperate of patients	26.99 (31.78)	77.04 (20.38)	< 0.001
Care of non -infected patients and non-transmitted diseases such as AIDS and hepatitis Care for infectious patients and fear of transmitted diseases such as AIDS and hepatitis	27.15 (30.91)	37 (26.81)	< 0.001
Adequate technical skills of nurses Inadequate technical skills of nurses	24.50 (29.80)	26.54 (28.11)	0.381
**Management dimension**	59.70 (17.75)	71.68 (11.90)	< 0.001
*Experienced teachers of professional ethics during education *Inexperienced teachers of professional ethics during education	81.25 (18.34)	83.40 (11.82)	0.044
*Receive the necessary training on ethical issues during your education *Failure to receive the necessary training on ethical issues during your education training​	79.31 (20.81)	81.25 (18.93)	0.089
*The existence of written policies or standards of nursing care legislation Absence of written policies or standards of nursing care legislation	70.90(31.40)	85.56 (21.45)	< 0.001
*Existence of in-service training and educational programs on professional ethics Absence of in-service training and educational programs on professional ethics	64.49 (42.28)	84.73 (15.23)	< 0.001
*Control and supervision of nursing managers in compliance with professional ethics Lack of control and supervision of nursing managers in accordance with professional ethics	63.82 (33.37)	83.35 (23.73)	< 0.001
*Moral and legal support of staff by senior managers Lack of moral and legal support of staff by senior managers	62.88 (36.90)	79.03 (20.22)	< 0.001
Proper work shifts (proportion between the working hours of the personnel and the number of shifts) Improper work shifts (disproportion between the working hours of the personnel and the number of shifts)	68.25 (34.44)	75.82 (24.02)	0.006
Sufficient number of personnel Inadequate number of personnel	68.14 (42.59)	73.94 (30.33)	< 0.001
Paying attention to the training needs of personnel and planning to improve meeting needs Failure to pay attention to the training needs of personnel and planning to meet the needs	47.17 (33.57)	71.62 (29.47)	< 0.001
Long shift Short shift	48.61(32.44)	62.61 (30.17)	< 0.001
*Existence of codes of ethics Absence of codes of ethics	62.55 (37.57)	80.64 (21.41)	< 0.001
Proper relationship between supervisors and personnel Inappropriate relationship between supervisors and personnel	37.72 (27.38)	60.50 (31.76)	< 0.001
Management of crisis in the ward Lack of crisis management in the department	51.38 (30.41)	52.76 (30.29)	< 0.001
Paying attention to staff’s abilities and skills in the division of duties Not Paying attention to staff’s abilities and skills in the division of duties	29.25 (31.54)	28.20 (32.76)	0.688
**Environmental dimension**	44.67 (15.29)	44.67 (15.29)	1
*Fixed shift Rotational shift works	60.01 (36.33)	79.59 (25.35)	< 0.001
No negative effect of night shift on health Negative effects of night shift on health	72.89 (26.02)	77.10 (25.92)	< 0.001
Reasonable expectations of patients and their caregivers from staff Unreasonable expectations of patients and their caregivers from staff	26.93 (31.15)	74.17 (22.22)	< 0.001
No overcrowding in ward Ward congestion	45.46 (30.36)	72.34 (27.68)	< 0.001
Adequacy of available facilities and equipment Insufficient available facilities and equipment	18.03 (27.22)	30.91 (30.23)	< 0.001

Note: The score range of the items is between 0-100 in each row

The first item in each row is facilitator and the second item is inhibitor to compliance with PES

SD: Standard Deviation, PES: Professional Ethics Standards

^a^ According to Wilcoxon signed ranks test

*The most important inhibitors and facilitators of compliance with PES

## Discussion

Based on the results of the present study, the “Individual care-related” dimension in the role of facilitator was the most important dimension from the nurses’ perspective in complying with professional ethics. Additionally, seven items related to both facilitating and inhibiting roles had the same importance which are as follows: strong or weak belief or disbelief in compliance with ethical issues, having or not having knowledge and awareness about the principles of professional ethics, ability or inability to critical thinking or ethical decision-making, high or low workload and its impact on accuracy and concentration and its effect on the accuracy and concentration of nurses, experienced or inexperienced teachers of professional ethics during education, effective and appropriate or ineffective and inappropriate communication with the patients and experienced or in experienced teachers of professional ethics during education. In the mentioned items, even though the comparison of the mean score of each item in two facilitating and inhibiting roles showed that one of them was significantly greater, due to the closeness of their scores to each other and the high score of both, the importance of both facilitating and inhibiting roles were interpreted similarly.

“Strong or weak belief in compliance with ethical issues” was an important antecedent in compliance with PES from the nurses’ perspective. In fact, nurses’ individual beliefs can have direct and indirect dual effects on ethical behavior. In other words, the behavior of nurses is a direct manifestation of their belief content to comply with the standards of professional ethics, and its manifestation in the behavior of other nurses and nursing students is an indirect manifestation of the ethical beliefs of individuals. Shafakhah et al. state that the practical manifestation of nurses’ beliefs can be a model for the performance of others and the main driver for the development of beliefs such as responsibility, correct communication, altruism, and respect for patient privacy and confidentiality in other colleagues and nursing students [13].

In Saeeidi et al.’s study, the strong beliefs and religious foundations of nurses in terms of professional and humane duties were found to be important facilitators of compliance with PES [8]. However, in Dehghani et al.‘s study, which was conducted with the aim of determining the barriers to complying with PES, “weakness of belief toward compliance with ethical issues” was not a significant inhibitor from the nurses’ perspective [10]. Considering that the present study is the first to investigate the importance of this subject in compliance with professional ethics in both facilitating and inhibiting roles from the nurses’ perspective, it can be inferred that “having a strong belief in compliance with the ethics” is as important as “weakness of belief toward ethical compliance with” for nurses, so they pay attention to both facilitating and inhibiting roles of this factor.

The other important inhibitor and facilitator of compliance with PES from the nurses’ perspective was “knowledge and awareness about the principles of professional ethics”. Nasiriani et al. showed that the high ethical knowledge of nurses causes patients’ rights to be respected [14]. According to Dehghani et al.’s study, 55.3% of nurses considered a “lack of knowledge and awareness about the principles of professional ethics” to be a significant inhibitor in complying with PES [10], which is consistent with the results of our study, and the importance of each role of facilitation and inhibition makes this factor more prominent from the nurses’ perspective.

The next factor is ability or inability to engage in critical thinking or ethical decision-making in the dual role of facilitator and inhibitor. In fact, nurses use professional ethics codes during the systematic process of critical thinking to make the best ethical decisions, and this is a factor in complying with PES [15]. According to Esmaelzadeh et al., the ethical decision-making ability of nurses can lead to ethical care [9]. Additionally, Taheri et al. stated that nurses’ inability to make ethical decisions is the most important inhibitor in compliance with PES [16], which points to the dual nature of the role of this item. This finding is consistent with the results of our study and shows that nurses care about both the facilitating and inhibiting roles of this item.

“Workload and its impact on the accuracy and concentration of nurses” was the other significant inhibitor and facilitator of complying with PES from the nurses’ perspective. This study showed that the high workload of nurses as an inhibitor and the low workload of nurses as a facilitator are almost equally important from the nurses’ perspective in complying with PES. Haahr et al. consider the high workload of nurses to an important inhibitor of compliance with professional ethics and state that nurses have likened the ability to manage workload to a business, which needs to perform a high workload within a time limit, the presence of mind, and high concentration, so they will be able to pay attention to ethical issues related to caring for subjects at the same time. He believes that factors such as lack of personnel, time constraints, and high workload in the managerial dimension are inhibitors in the management of nursing practice in the individual dimension [3].

“Effective and appropriate or in effective and inappropriate communication with the patients” is another significant antecedent in complying with PES from the nurses’ perspective, in such a way that proper nurse‒patient communication facilitates patients’ participation in care decisions, and in this way, compliance with PES, such as autonomy and nonmaleficence, is facilitated [17].

The ‘’experienced or in experienced teachers of professional ethics during education’’ is one of the most important inhibitors and facilitators of compliance with professional ethics. Shafakhah et al. and Borhani et al. stated that ethics lecturers, in addition to having an educational role in ethical and legal nursing issues, play an ethical role model for nursing students. Therefore, students’ personality characteristics, beliefs, religious beliefs, honesty, and accountability will be practical models for them, and they will improve their knowledge and awareness about ethical issues [13, 18].

Our study showed that one side of the spectrum of the antecedents of PES compliance (inhibiting-facilitating spectrum) may be more important than the other side from the nurses’ perspective. In this way, not only were the mean scores of the facilitating part significantly higher than those of the inhibiting part, but the mean differences between the two facilitating and inhibiting parts of those items were also greater. Therefore, in the case of some factors, the nurses’ perspective focused on the facilitating section.

For example, nurses’ attention to the items “income sufficiency or adequate rest in nursing staff” and ‘’fixed shift’’ as facilitators shows that inadequate income and insufficient rest and rotational shifts (physiological rhythm disruptors) are not important inhibitors of compliance with PES. This is contrary to Maslow’s levels of needs and shows that inadequate income and insufficient rest as physiological needs are important inhibitors of compliance with PES at the level of self-actualizer needs [19]. In the studies of Asadi et al. and Dehghani et al., sufficient income from nurses and fixed shifts was also reported to be an effective step in compliance with professional ethics [10, 20]. Its causes may lie in religious beliefs, which cause nurses to consider the observance of ethical principles before their physical needs. For example, in Iran, which is religious Islam, nurses consider following religious laws (the right of the people takes precedence over their own right) to be more involved in their care activities [21].

Three other facilitating factors, ‘’the existence of written policies or standards of nursing care legislation’’, ‘’existence of in-service training, educational programs on professional ethics’’ and ‘’sufficient previous familiarity with the skills’’, can be related to the cognitive ability of nurses, and their absence was not very important as an inhibitor from nurses’ perspective. The reason for this can be related to the use of substitutes for each of these factors, such as their previous knowledge, individual experiences, asking colleagues, referring to scientific sources and personal and religious beliefs.

Also “control and supervision of nursing managers in compliance with professional ethics” and “moral and legal support of staff by senior managers” are factors that are affected by organizational aspects according to Pennino [22]. Additionally, Douglas et al. emphasized that if the managers of an organization apply ethical principles in the work environment, an ethical atmosphere prevails in the organization, which can surround other staff in the organization [23]. According to our review of the literature, the reasons why nurses focus only on the facilitating role of these factors are undiscovered, and more studies need to be conducted in this field.

Furthermore, the item “existence of codes of ethics” was a significant facilitator of compliance with PES. Because nurses are in constant contact with patients, they face many challenges that require ethical decision-making [18], and ethical decision-making requires the use of professional ethics codes as rules and guidelines [15]. Therefore, from the point of view of nurses, the presence of codes of ethics as a facilitator on one side of the scale of ethical decision-making is heavier than that on the other side of the scale in the form of the “absence of codes of ethics” as an inhibitor.

The low mean score (less than 50) for some items and dimensions, such as “adequate or inadequate technical skills of nurses, paying or not paying attention to staff’s abilities and skills in the division of duties and environmental dimension” of complying with the PES, showed that although these items are not unimportant from the nurses’ perspective, they are less important than items with a high mean score in complying with the PES.

Finally, it can be said that finding the root causes of this attention to one side of the inhibitory-facilitative spectrum requires conducting qualitative studies in the future.

The participants’ answers to the “open-ended question” section revealed new factors that inhibited and facilitated compliance with PES. The “too many works shift due to forced overtime, lack of encouragement and punishment criteria for compliance with professional ethics, and lack of peace in the work environment” have been the inhibitors to compliance with PES from the nurses’ perspective, which can be considered an unfavorable work environment.

An unfavorable work environment leads to poor interpersonal relationships, lack of altruism in care, dissatisfaction with the workplace, insufficient knowledge, disregard for patients’ privacy, provision of one-dimensional nursing care, and weakness in accepting professional responsibility [13]. According to nurses, empathy and compassion toward patients were facilitating factors in this part of the study. It can be rooted in the religious culture and spirituality of Iranian nurses in such a way that it causes internalization of the factor of professional commitment (Ibid). The fatigue caused by working with masks and personal protective equipment (PPE) during the COVID-19 pandemic was another inhibitor in compliance with professional ethics. According to Silverman et al., the strategy of using personal protective equipment is an inhibitor in nurses’ caring role and is considered one of the main causes of nurses’ dissatisfaction [24].

## Conclusion

Our study showed that nurses can have three orientations regarding the factors that are effective in complying with PES in terms of importance. In such a way, both the facilitating and inhibiting roles of effective factors in complying with PES may be more or less important, or only the facilitating role of some of these factors may be more prominent for them. It is recommended that future studies, especially qualitative studies, be conducted to find the causes of this triple orientation from the nurses’ perspective in complying with PES.

### Limitations

One of the limitations of this study is the possibility of generalizing its results to other populations because it was only conducted in hospitals in one city. Therefore, it is suggested that the importance of the facilitating and inhibiting role of these factors be investigated from the nurses’ perspective in other nurses’ communities.

### Implications for practice

The findings of this study can be used in nursing practice and research. Awareness of the facilitators or inhibitors of PES compliance from the nurses’ perspective reveals the opportunities and threats to PES compliance and can be used as a guide for nurses to promote compliance with PES in nurses and help to improve the quality of nursing care. Considering that in the current study, individual beliefs were the most important antecedent in complying with PES, there is a need to examine individual beliefs related to ethical issues among applicants entering the nursing profession. It is also suggested that future studies be conducted in the field of designing scales to examine nurses’ individual beliefs in ethical compliance. Since some of the effective factors in compliance with professional ethics, nurses were interested only in facilitating compliance with professional ethics; therefore, it is suggested that qualitative studies be conducted to explore the root causes.

### Electronic supplementary material

Below is the link to the electronic supplementary material.

Supplementary Material 1

## Data Availability

The datasets used and analyzed during the current study are available from the corresponding author upon reasonable request.

## Abbreviations

PES: Professional Ethics Standards
SD: Standard Deviation
