# Supplementary material for: Inhibitors and facilitators of compliance with professional ethics standards: nurses’ perspective
Source: BMC Nurs. 2024 Mar 5;23:158. doi: 10.1186/s12912-024-01829-9 (PMC10913360; doi:10.1186/s12912-024-01829-9)
Supplement: Supplementary file 1 — Supplementary Material 1 [file 12912_2024_1829_MOESM1_ESM.docx]

"Inhibitors of compliance with professional ethics standards by the nurses' perspective" questionnaire

| Domain | # | Item | Completely agree | Agree | no opinion | disagree | strongly disagree |
| --- | --- | --- | --- | --- | --- | --- | --- |
| Management | 1 | Not Paying attention to staff’s abilities and skills in the division of duties |  |  |  |  |  |
|  | 2 | Lack of control and supervision of nursing managers in accordance with professional ethics |  |  |  |  |  |
|  | 3 | Failure to pay attention to the training needs of personnel and planning to meet the needs |  |  |  |  |  |
|  | 4 | Absence of codes of ethics |  |  |  |  |  |
|  | 5 | Lack of crisis management in the department |  |  |  |  |  |
|  | 6 | Improper work shifts (disproportion between the working hours of the personnel and the number of shifts) |  |  |  |  |  |
|  | 7 | Inadequate number of personnel |  |  |  |  |  |
|  | 8 | Long shift |  |  |  |  |  |
|  | 9 | Inappropriate relationship between supervisors and personnel |  |  |  |  |  |
|  | 10 | Absence of in-service training and educational programs on professional ethics |  |  |  |  |  |
|  | 11 | Absence of written policies or standards of nursing care legislation |  |  |  |  |  |
|  | 12 | Lack of experience of professional ethics teachers with experience during education |  |  |  |  |  |
|  | 13 | Failure to get the necessary training on ethical issues during my education |  |  |  |  |  |
|  | 14 | Lack of moral and legal support of staff by senior managers |  |  |  |  |  |
| Environmental | 15 | Insufficient available facilities and equipment |  |  |  |  |  |
|  | 16 | Negative effects of night shift on health |  |  |  |  |  |
|  | 17 | Ward congestion |  |  |  |  |  |
|  | 18 | Rotational shift works |  |  |  |  |  |
|  | 19 | Unreasonable expectations of patients and their caregivers from staff |  |  |  |  |  |
| Individual care-related | 20 | Not having knowledge and awareness about the principles of professional ethics |  |  |  |  |  |
|  | 21 | Not having enough time required to perform care |  |  |  |  |  |
|  | 22 | Carrying out completely new tasks that she encounters for the first time and does not have sufficient skills about |  |  |  |  |  |
|  | 23 | High workload and its impact on accuracy and concentration of nurses |  |  |  |  |  |
|  | 24 | Inappropriate behaviors of patients with nursing staff (communication with patients who do not cooperate) |  |  |  |  |  |
|  | 25 | Negative attitude to professional ethics standards in nursing |  |  |  |  |  |
|  | 26 | Care for infectious patients and fear of transmitted diseases such as AIDS and hepatitis |  |  |  |  |  |
|  | 27 | Lack of interest and motivation to the profession in nursing personnel |  |  |  |  |  |
|  | 28 | Failure to respond to basic needs such as adequate income or rest in nursing staff |  |  |  |  |  |
|  | 29 | Dissatisfaction with the work place |  |  |  |  |  |
|  | 30 | Inadequate technical skills of nurses |  |  |  |  |  |
|  | 31 | Inability to critical thinking or ethical decision-making |  |  |  |  |  |
|  | 32 | In effective and inappropriate communication with the patient |  |  |  |  |  |
|  | 33 | Weak belief in compliance with ethical issues |  |  |  |  |  |

"Facilitators of compliance with professional ethics standards by the nurses’ perspective" questionnaire

| Domain | # | Item | Completely agree | Agree | No opinion | disagree | Strongly disagree |
| --- | --- | --- | --- | --- | --- | --- | --- |
| Management | 1 | Paying attention to staff’s abilities and skills in the division of duties |  |  |  |  |  |
|  | 2 | Control and supervision of nursing managers in compliance with professional ethics |  |  |  |  |  |
|  | 3 | Paying attention to the training needs of personnel and planning to improve meeting needs |  |  |  |  |  |
|  | 4 | Existence of codes of ethics |  |  |  |  |  |
|  | 5 | Management of crisis in the ward |  |  |  |  |  |
|  | 6 | Proper work shifts (proportion between the working hours of the personnel and the number of shifts) |  |  |  |  |  |
|  | 7 | Sufficient number of personnel |  |  |  |  |  |
|  | 8 | Short shift |  |  |  |  |  |
|  | 9 | Proper relationship between head nurses and personnel |  |  |  |  |  |
|  | 10 | Existence of in-service training and educational programs on professional ethics |  |  |  |  |  |
|  | 11 | The existence of written policies or standards of nursing care legislation |  |  |  |  |  |
|  | 12 | The experience of professional ethics teachers during education |  |  |  |  |  |
|  | 13 | Get the necessary training on ethical issues during your education |  |  |  |  |  |
|  | 14 | Moral and legal support of staff by senior managers |  |  |  |  |  |
| Environmental | 15 | Adequacy of available facilities and equipment |  |  |  |  |  |
|  | 16 | Reducing the negative effect of night shifts on the health. (Adequate rest in the night shifts) |  |  |  |  |  |
|  | 17 | No overcrowding in ward |  |  |  |  |  |
|  | 18 | Fixed shift works |  |  |  |  |  |
|  | 19 | Educate the patient about their expectations of the nursing staff (Reasonable expectations of patients and their caregivers from staff) |  |  |  |  |  |
| Individual care-related | 20 | Having knowledge and awareness about the principles of professional ethics |  |  |  |  |  |
|  | 21 | Having enough time required to perform care |  |  |  |  |  |
|  | 22 | Sufficient previous familiarity with the skills she is performing for the first time |  |  |  |  |  |
|  | 23 | Low workload and its impact on accuracy and concentration of nurses |  |  |  |  |  |
|  | 24 | Appropriate behaviors of patients with nursing staff (communication with patients who cooperate) |  |  |  |  |  |
|  | 25 | Positive attitude to professional ethics standards in nursing |  |  |  |  |  |
|  | 26 | Care of non -infected patients and transmitted diseases such as AIDS and hepatitis |  |  |  |  |  |
|  | 27 | Interest and motivation towards the profession in nursing personnel |  |  |  |  |  |
|  | 28 | Responding to basic needs such as income sufficiency or adequate rest in nursing staff |  |  |  |  |  |
|  | 29 | Satisfaction with the work place |  |  |  |  |  |
|  | 30 | Adequate technical skills of nurses |  |  |  |  |  |
|  | 31 | Ability to critical thinking or ethical decision-making |  |  |  |  |  |
|  | 32 | Effective and appropriate communication with the patient |  |  |  |  |  |
|  | 33 | Strong belief in compliance with ethical issues |  |  |  |  |  |

*Please share with the research team other factors of compliance with professional ethics that were not mentioned in the questionnaire.

………………………………………………………………………………………………………………………………………………………………………………………………………………………………………………………………………………………………………
